# Tumor Necrosis Factor-Alpha and Adiponectin in Nonalcoholic Fatty Liver Disease-Associated Hepatocellular Carcinoma

**DOI:** 10.3390/cancers15215306

**Published:** 2023-11-06

**Authors:** Ilias D. Vachliotis, Ioannis Valsamidis, Stergios A. Polyzos

**Affiliations:** 1 First Laboratory of Pharmacology, School of Medicine, Aristotle University of Thessaloniki, 54124 Thessaloniki, Greece; ivachliot@auth.gr; 2 Department of Gastroenterology, 424 General Military Hospital, 56429 Thessaloniki, Greece; 3 First Department of Internal Medicine, 424 General Military Hospital, 56429 Thessaloniki, Greece; jonvalsamidis87@gmail.com

**Keywords:** hepatocellular carcinoma, nonalcoholic fatty liver disease, nonalcoholic steatohepatitis, adiponectin, tumor necrosis factor-alpha

## Abstract

**Simple Summary:**

The prevalence of nonalcoholic fatty liver disease (NAFLD)-associated hepatocellular carcinoma (HCC) is rapidly increasing, following the growing prevalence of NAFLD. The pathophysiological mechanisms of NAFLD-associated HCC are not fully elucidated. Emerging preclinical and limited clinical studies suggest that tumor necrosis factor-α (TNF-α) and adiponectin may contribute to the progression from NAFLD to HCC. This may render both TNF-α and adiponectin as appealing therapeutic targets in the setting of NAFLD-associated HCC, for which systemic immunotherapy, i.e., immune checkpoint inhibitors (ICIs), seems to be less effective compared to HCC of other etiologies. Therefore, anti-TNF biologics and/or adiponectin analogs or medications that increase endogenous adiponectin may be investigated combined with ICIs in the treatment of NAFLD-associated HCC.

**Abstract:**

Nonalcoholic fatty liver disease (NAFLD) is emerging as an important risk factor for hepatocellular carcinoma (HCC), whose prevalence is rising. Although the mechanisms of progression from NAFLD to HCC are not fully elucidated, tumor necrosis factor-α (TNF-α) and adiponectin, as well as their interplay, which seems to be antagonistic, may contribute to the pathophysiology of NAFLD-associated HCC. TNF-α initially aims to protect against hepatocarcinogenesis, but during the progression of NAFLD, TNF-α is increased, thus probably inducing hepatocarcinogenesis in the long-term, when NAFLD is not resolved. On the other hand, adiponectin, which is expected to exert anti-tumorigenic effects, is decreased during the progression of the disease, a trend that may favor hepatocarcinogenesis, but is paradoxically increased at end stage disease, i.e., cirrhosis and HCC. These observations render TNF-α and adiponectin as potentially diagnostic biomarkers and appealing therapeutic targets in the setting of NAFLD-associated HCC, possibly in combination with systematic therapy. In this regard, combination strategy, including immune checkpoint inhibitors (ICIs) with anti-TNF biologics and/or adiponectin analogs or medications that increase endogenous adiponectin, may warrant investigation against NAFLD-associated HCC. This review aims to summarize evidence on the association between TNF-α and adiponectin with NAFLD-associated HCC, based on experimental and clinical studies, and to discuss relevant potential therapeutic considerations.

## 1. Introduction

Nonalcoholic fatty liver disease (NAFLD) is emerging as the most prominent chronic liver disease worldwide, affecting about 30% of the adult general population [1]. Notably, this rate is predicted to rise to 50% at least in Europe, the USA, and China within the next decade [2]. NAFLD is moving in parallel with obesity and type 2 diabetes mellitus (T2DM), in which its prevalence is even higher (50–90%). Among patients with NAFLD, about 20% and 7% are estimated to have nonalcoholic steatohepatitis (NASH) and advanced fibrosis, respectively, which represent more severe phenotypes of the disease [3]. NASH and hepatic fibrosis are associated with an increased risk of progression to cirrhosis and hepatocellular carcinoma (HCC), as well as cardiovascular diseases and extrahepatic malignancies [4].

HCC is the most common primary liver cancer with a 5-year survival rate estimated to be 18%, ranking HCC as the third leading cause of cancer-related mortality worldwide [5]. NAFLD has become an emerging risk factor for HCC; thus, the latter is expected to further increase in prevalence owing to the growing prevalence of NAFLD [6]. Intriguingly, NAFLD-associated HCC appears to differ in pathogenesis and responds differently to treatment compared with HCC of other etiologies, possibly due to specific pathophysiological mechanisms related to NAFLD [6,7].

Dysregulation of cytokines, such as tumor necrosis factor-α (TNF-α), and of adipokines, such as adiponectin, regarded as a key proinflammatory cytokine and adipokine, respectively, is implicated in the pathogenesis of NAFLD and possibly in the progression to HCC [8,9,10]. A comprehensive understanding of their potential contribution to NAFLD-associated HCC and their interplay may be essential for the possible future development of novel therapeutic strategies targeting NAFLD and NAFLD-associated HCC.

This review aimed to summarize current knowledge regarding TNF-α and adiponectin in NAFLD-associated HCC. The first two sections are devoted to the association between TNF-α and adiponectin with NAFLD-associated HCC, based, first, on experimental studies and, second, on clinical studies. Subsequently, potential therapeutic considerations relevant to TNF-α and adiponectin in NAFLD-associated HCC are discussed.

## 2. TNF-α and NAFLD-Associated HCC

### 2.1. Experimental Studies

TNF-α is a principal proinflammatory cytokine, regarded as an important contributor to the pathogenesis of NAFLD and its transition to NASH and possibly to advanced disease [10,11,12]. In NAFLD, TNF-α may originate from several sources: the dysfunctional adipocytes, lipid-stressed hepatocytes, and various immune cells, including both innate immune cells (Kupffer cells (KCs), myeloid-derived monocytes, and neutrophils) and adaptive immune cells (i.e., natural killer (NK)T cells, CD8+, and CD4+ Th1 lymphocytes) that infiltrate the steatotic liver [13,14]. Notably, a distinct subset of resident CXCR6+ PD1+ TNF+ CD8+ T cells was discovered, which is assumed to play an important role in the transition of simple steatosis (nonalcoholic fatty liver; NAFL) to NASH and HCC [15]. These cells exhibit an hyperactive state known as “auto-aggression” [16], recognize metabolic-associated signals (e.g., acetate, P2RX7 receptor ligands), and react to metabolic stimuli by secreting large amounts of TNF-α [17]. After its production, TNF-α acts via its two cognate receptors, tumor necrosis factor receptor (TNFR)1 and TNFR2, and activates the nuclear factor-kappa B (NF-κΒ), a transcription factor that is considered the primary downstream effector of TNF-α [18]. It is highlighted that NF-κΒ mediates inflammation, but also the survival of the hepatocytes [19]. Thus, the role of NF-κΒ is complex and seems to be dual in hepatocarcinogenesis. On the one hand, basal NF-κΒ activity targets the survival of hepatocytes by downregulating apoptotic genes; however, it should be underlined that hepatocyte apoptosis leads to the counteracting proliferation of the adjacent, healthy hepatocytes, thus increasing the risk of oncogenic mutations that induce hepatocarcinogenesis [20]; therefore, by preventing hepatocyte apoptosis, NF-κΒ seems to protect against hepatocarcinogenesis, which seemingly is a paradox. On the other hand, the activation of NF-κΒ links inflammation with tumorigenesis; thus, it promotes tumorigenesis by upregulating inflammatory genes [20]. Thus, it seems that, under basal circumstances, NF-κΒ may prevent hepatocarcinogenesis; however, when it is overactivated (e.g., by TNF-α), it may contribute to hepatocarcinogenesis. However, TNF-α may induce hepatocyte death (apoptosis or necroptosis) via another pathway, the assembly of intracellular protein adaptors, known as the death-inducing signaling complex (DISC) II [21]. Notably, NF-κΒ induces inhibitors of the DISC II signaling [21]; therefore, it seems that TNF-α may induce apoptosis via the activation of DISC II signaling, whereas it may inhibit apoptosis via the activation of NF-κΒ signaling, which cross-talks with DISC II signaling. In other words, TNF-α triggers inflammation, but also it plays a central role in the fate of hepatocytes, leading to either hepatocyte survival or hepatocyte death depending on certain circumstances, which are not fully elucidated [22]. Considering the above facts, in the setting of NAFLD, apoptosis of damaged hepatocytes is coupled with a counteracting proliferation of the adjacent, healthy hepatocytes, which, as mentioned above, may increase the risk of DNA damage and, subsequently, the risk of oncogenic mutations and malignant transformation of the hepatocytes. Additionally, TNF-α was shown to promote proliferation and differentiation of hepatic progenitor cells to malignant cells in diethyl-nitrosamine (DEN)-induced HCC rats, mainly through binding of TNFR2 [23]. Nevertheless, this additional mechanism of hepatocarcinogenesis should also be validated in NASH mouse models, because DEN is a chemical carcinogen that causes HCC in mice through different mechanisms than high fat diet (HFD) and obesity [24]. Furthermore, TNF-α activates the c-Jun N-terminal kinase (JNK) pathway in hepatocytes, which is another intracellular signaling pathway commonly activated in HCC [25]. This specific pathway is induced by TNF-α, e.g., in the presence of lipotoxicity [22].

**Figure 1:**
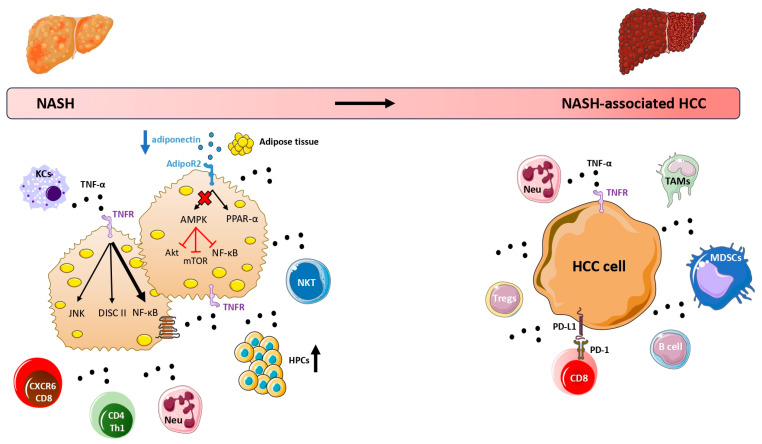
The potential contribution of TNF-α and adiponectin to the pathogenesis of NAFLD-associated HCC. In NASH, TNF-α is produced by dysfunctional adipocytes, lipid-stressed hepatocytes, and various immune cell populations that reside in the liver (KCs) or infiltrate the liver, including myeloid-derived monocytes, neutrophils, NKT cells, CXCR6+ PD1+ TNF+ CD8+ T cells, and CD4+ Th1 cells. TNF-α activates NF-κB, which, although under basal condition may prevent hepatocarcinogenesis, when overactivated, as in the setting of NASH, may contribute to hepatocarcinogenesis. In addition, TNF-α promotes hepatocyte death, which is mediated by the DISC II. Also, TNF-α activates specific tumor-associated pathways (i.e., the JNK pathway) in affected hepatocytes and promotes the proliferation and differentiation of HPCs, all of which contribute to hepatocarcinogenesis. In NASH-associated HCC microenvironment, TNF-α promotes the accumulation of several immune-suppressive cells, such as neutrophils, TAMs, MDSCs, Tregs, and B cells, which impair the function of anti-tumor CD8+ T cells. In addition, TNF-α induces the expression of PD-L1 by tumor cells, which interacts with PD-1 expressed on anti-tumor CD8+ T cells, allowing them to evade the immune system. Contrary to TNF-α, adiponectin, being considered beneficial to NAFLD, is diminished in NAFLD; thus, its low concentrations may promote NASH and possibly hepatocarcinogenesis. In the liver, adiponectin acts predominantly through AdipoR2 and activates the AMPK and PPAR-α pathways, which leads to insulin-sensitizing, anti-steatotic, and anti-inflammatory effects. Importantly, AMPK activation seems to be critical for the inhibition of the mTOR, NF-κB, and Akt downstream pathways, which all contribute to the potentially anti-tumorigenic properties of adiponectin. In other words, low adiponectin in NASH results in the decreased activation of AMPK and the subsequent stimulation of tumor-promoting pathways in lipid-stressed hepatocytes. Abbreviations: AdipoR2, adiponectin receptor 2; AMPK; adenosine monophosphate-activated protein kinase; DISC, death-inducing complex; HCC, hepatocellular carcinoma; HPCs, hepatic progenitor cells; JNK, c-Jun N-terminal kinase; KCs, Kupffer cells; mTOR, mammalian target of rapamycin; MDSCs, myeloid-derived suppressor cells; NAFLD, nonalcoholic fatty liver disease; NASH, nonalcoholic steatohepatitis; NF-κΒ, nuclear factor-kappa B; PPAR, peroxisome proliferator-activated receptors; PD-1, programmed death-1; PDL-1, programmed death-ligand 1; TAMs, tumor-associated macrophages; TNF-α, tumor necrosis factor alpha; Tregs, T regulatory cells; ↑, increase; and ↓, decrease.

Summarizing the above facts, TNF-α appears to be associated with increased hepatocellular death that increases the likelihood of oncogenic mutations, as well as the activation of mitogenic pathways (i.e., the JNK signaling), in the presence of lipotoxicity, all of which possibly link TNF-α to HCC in the context of long-term and unresolved NAFLD, which is associated with a low-grade, but chronic intrahepatic inflammation [26]. We previously supported a dual-faceted effect of adipokines and cytokines in NAFLD [27]. In this regard, we may hypothesize that TNF-α initially induces inflammation by activating NF-κΒ, as a counteracting mechanism against hepatic steatosis; this mechanism initially seems to protect against hepatocarcinogenesis (through NF-κΒ signaling). However, when this mechanism fails to resolve NAFLD, the long-term activation of TNF-α shifts to be disadvantageous for NAFLD, by preserving intra-hepatic inflammation in the long-term; this state is associated with hepatocarcinogenesis via the activation of other signaling pathways, e.g., DISC II and JNK (Figure 1). However, this hypothesis remains to be proven.

Once HCC is developed in the setting of NAFLD, the tumor microenvironment is characterized by a sophisticated communication network between tumor cells and immune cells, which is also influenced by the local and systemic, metabolic and inflammatory dysfunction [7,28]. This crosstalk between metabolic signals, inflammatory mediators, tumor cells, and immune cells appears to promote an immune-suppressive microenvironment, where several suppressive cells, such as neutrophils, tumor-associated macrophages (TAMs), myeloid-derived suppressor cells (MDSCs), regulatory T cells (Tregs), and B cells infiltrate into the tumor and impair the anti-tumor surveillance properties of CD8+ T cells, the main cytotoxic cells against malignant hepatocytes [29]. In this micromilieu, TNF-α appears to play key roles. TNF-α promotes the accumulation of MDSCs in tumor tissue, which are considered important negative immune regulators [30]. In addition, TNF-α derived from TAMs facilitates the epithelial-to-mesenchymal transition (EMT) of HCC cells, which enables them to evade the immune system by increasing the expression of programmed death-ligand 1 (PD-L1) [31,32]. Notably, PD-L1, expressed by tumor cells, interacts with PD-1 receptors on CD8+ T cells, thus suppressing their activity [33]; it is underlined that the interaction of PD-L1/PD-1 is a pharmacological target of immunotherapy against many types of cancer, including HCC [34]. Furthermore, TNF-α derived from adipocytes was also shown to induce PD-L1 expression in HCC tumor cells of obese mice, which may mechanistically link obesity with the progression of NAFLD-associated HCC [35]. Hence, TNF-α induces processes and mechanisms that may favor both the development and progression of HCC in NAFLD (Figure 1). Nevertheless, it remains uncertain whether TNF-α plays a more significant role in early stages (i.e., development of HCC) or advanced stages (i.e., progression of HCC), which warrants further investigation.

### 2.2. Clinical Studies

Observational studies suggest that TNF-α is associated with NAFLD severity [36]; in this regard, a meta-analysis of observational studies showed that circulating TNF-α concentrations gradually increase from non-NAFLD to NAFL and then to NASH patients [37]. Similarly, we have recently reviewed the role of TNF-α in the pathogenesis and treatment of NAFLD, supporting an association between higher TNF-α and the severity of the disease, which may have therapeutic implications [10]. In addition, obesity, especially visceral obesity that is present in most patients with NAFLD, is known to be a state of subclinical chronic inflammation, which is characterized by elevated production and secretion of inflammatory cytokines, including TNF-α. Notably, it seems that adipose tissue-derived cytokines may promote hepatocarcinogenesis independent of NAFLD [38]. Indeed, the development of DEN-induced liver tumors in mice was accelerated by obesogenic diet or genetic obesity, an effect that depended on the oncogenic signaling pathways mediated by adipose-derived TNF-α and IL-6 [39]. Although more relevant data are needed, it seems that TNF-α may mediate, at least partly, the effect of obesity in the progression of NAFLD, including NAFLD-associated HCC.

Nonetheless, the exact role of TNF-α in HCC and, more specifically, in NAFLD-associated HCC, remains largely understudied, which is consistent with our knowledge gaps pertaining to the mechanisms of progression from NASH or liver fibrosis/cirrhosis to HCC [6]. To date, only one study showed that circulating TNF-α and hepatic TNF-α mRNA were gradually increased from the controls to patients with NASH and then to patients with NAFLD-associated HCC [40]. Other studies, which are also limited in number, have reported associations between TNF-α and HCC without specifying the underlying cause of HCC or by referring to HCC of different etiologies; therefore, their results, which are summarized hereby, should be cautiously extrapolated specifically to patients with NAFLD-associated HCC. A study of 31 males with cirrhosis and HCC, 26 males with cirrhosis without HCC, and 25 controls showed that circulating TNF-α was higher in cirrhosis and HCC compared with controls, but did not differ between cirrhotic patients with and without HCC [41]. However, when patients with HCC were classified according to tumor stage (based on Okuda’s classification [42]), circulating TNF-α was higher in more advanced stages of the disease [41]. On the contrary, a study of 97 HCC patients showed that patients with advanced disease (stage III and IV, according to the TNM classification [43]) did not have a higher expression of TNF-α in HCC tumor samples compared with those with TNM stage I and II [44]. However, survival analysis in this study showed that the overall survival was shorter in HCC patients with a higher TNF-α hepatic expression versus those with a lower TNF-α expression [44]. Nonetheless, a study of 83 HCC patients with early-stage HCC (TNM stage I) showed that patients with a higher TNF-α expression in tumor tissue had a lower risk of recurrence and mortality compared with those with a lower TNF-α expression [45]. Similarly, another study recruiting HCC patients with early or intermediate disease (Barcelona Clinic Liver Cancer-0-B (BCLC-0-B) [46]) also showed that a higher TNF-α expression in tumor tissue was associated with a better postoperative survival [47].

Moreover, two meta-analyses also favored the potential association between TNF-α and TNFR2 with HCC, although these meta-analyses did not include studies referring only to NAFLD-associated HCC populations. In one of them, specific single nucleotide polymorphisms (SNPs) (−863 C/A, −857 C/T, −308 G/A, and −238 G/A) on TNF-α gene promoter were associated with an increased risk of HCC, possibly through the upregulation of TNF-α expression, thus highlighting that TNF-α might be an important risk factor of HCC [48]. In the other meta-analysis, higher concentrations of soluble TNFR2 were associated with a higher risk of various types of cancer, showing the strongest association with HCC [49]. Notably, TNFR2 is highly expressed in the tumor microenvironment mainly by Tregs, playing a key role in their activation and expansion [50]. In addition, *tnfr2* gene is also expressed by tumor cells, in which it functions as an oncogene critically implicated in the pathogenesis of tumorigenesis [51].

Collectively, experimental evidence suggests that TNF-α is an important contributor to the pathogenesis of NAFLD-associated HCC. However, its exact role and utility in the clinical setting remain obscure due to the limited existing data. Based on the above-mentioned clinical evidence, which mainly refer to HCC of any etiology and not specifically to NAFLD-associated HCC, TNF-α may not be a suitable circulating biomarker for the early detection of HCC in cirrhotic patients, since its serum concentrations did not differ between cirrhotic patients with and without HCC. However, TNF-α, especially its expression in tumor tissue, may be more relevant to prognosis among patients with HCC, although existing data are inconsistent. Based on these data, we could speculate that a higher expression of TNF-α in HCC tissue may be associated with a better prognosis in early-stage HCC [45,47], but with worse prognosis in late-stage HCC [44]; obviously, this hypothesis remains to be proven in future studies. In addition, circulating TNF-α, which can be easily measured, may largely differ from tissue TNF-α, which requires liver biopsy. Furthermore, TNF-α expression may be different in the tumor tissue compared with the adjacent healthy liver tissue, which also should be proven, since it may bear pathophysiological and clinical implication. Therefore, more studies evaluating the role and utility of circulating, hepatic, and tumor TNF-α specifically in patients with NAFLD-associated HCC are warranted in the near future.

## 3. Adiponectin and NAFLD-Associated HCC

### 3.1. Experimental Studies

Adiponectin is a major adipokine secreted mainly by the adipocytes in large quantity [52]. Important insulin-sensitizing, anti-steatotic, and anti-inflammatory actions have been attributed to adiponectin, but its production is paradoxically diminished in obesity and NAFLD [53,54] (Figure 1). Contrary to TNF-α, which promotes the transition of NAFLD to HCC, adiponectin may exert potentially favorable effects on NAFLD and NAFLD-associated HCC; it seems that adiponectin prevents hepatic steatosis, inflammation, and possibly hepatic fibrosis, and it is evident that adiponectin antagonizes intracellular tumor-promoting pathways related to HCC cell proliferation, migration, and invasion, while it promotes HCC cell apoptotic pathways [55].

More specifically, adiponectin treatment induced apoptosis and inhibited the proliferation of HCC HepG2 and HuH7 cells lines in vitro, mainly by increasing the phosphorylation of adenosine monophosphate-activated protein kinase (AMPK), which subsequently led to the decreased activation of mammalian target of rapamycin (mTOR) [56]. Notably, adiponectin acts predominantly on the transmembrane adiponectin receptor (AdipoR)2 in the liver and activates the AMPK and peroxisome proliferator-activated receptor (PPAR)-α pathways [57]. AMPK activation seems to be critical for the activation of p53 and the inhibition of the mTOR, NF-κB, and Akt downstream pathways, which seem to partly contribute to the potential anti-tumorigenic properties of adiponectin [58]. In addition, hypoadiponectinemia with adiponectin knockout in mice with choline-deficient L-amino acid-defined (CDAA) diet-induced NAFLD enhanced the progression to NASH and hepatic tumor formation [59], which was also confirmed by a subsequent study with adiponectin knockout mice on high fat diet (HFD) mice [60]. Furthermore, the administration of adiponectin in thioacetamide-induced HCC rats, which, however, does not represent an experimental NAFLD model, reversed liver tumors, which was mechanistically linked to the induction of apoptotic pathways through the restoration of p53 and TNF-related apoptosis-inducing ligand (TRAIL) activities, while reducing JNK expression [61], which as mentioned above, is commonly activated in HCC [25]. Similarly, thioacetamide-induced HCC rats treated with adiponectin presented an 80% increase in survival rate, a 73% reduction in average number of liver nodules, 46% decrease in serum alpha-fetoprotein (AFP), as well as a reduced expression of tumor invasion markers, TNF-α and NF-κΒ [62]. It is noteworthy that adiponectin seems to exert antagonistic relationship with TNF-α in NAFLD, by inhibiting the synthesis and activity of each other [63]. However, it needs to be assessed whether the antagonistic roles of adiponectin and TNF-α continue to exist and to play a pathophysiological role during the progression of the disease to NAFLD-associated HCC (Figure 1); if this is validated, it may have certain preventive and therapeutic perspectives for NAFLD-associated HCC.

### 3.2. Clinical Studies

Most clinical evidence for adiponectin in human NAFLD supports that hypoadiponectinemia is associated with the progression from NAFL to NASH [64,65]. In particular, our meta-analysis of 27 studies including 1545 NAFLD patients and 698 controls showed that circulating adiponectin levels were decreased in NAFLD patients compared with controls, and further decreased in NASH versus NAFL patients [66], which, in fact, followed the opposite direction compared to TNF-α [37]. Intriguingly, circulating adiponectin seems to show a non-linear distribution in NAFLD, being low in NASH, but increases when NASH progresses to cirrhosis [67]. This was speculated to occur due to impaired hepatic and renal function, when NAFLD progresses to cirrhosis, but a dual effect of adiponectin cannot be excluded, i.e., adiponectin may shift from an advantageous to a disadvantageous contributor, when the disease progresses [67]. However, the role of adiponectin in NAFLD-associated HCC is largely unknown, owing to the limited available clinical studies. In the most relevant study to date of 191 patients with biopsy-proven NAFLD and advanced fibrosis (F3-F4) (119 without and 72 with NAFLD-associated HCC), it was shown that circulating adiponectin was higher in those with than without HCC [68]. In this study, adiponectin showed a similar performance to AFP (the latter considered to be a predictor of HCC [69]) for discriminating NAFLD patients with advanced fibrosis and HCC than those without HCC, independent of potential cofounders [68].

Other observational studies have attempted to investigate the association between adiponectin and HCC, but they have mainly focused on diverse populations of HCC patients and mostly viral-associated HCC patients and not particularly NAFLD-associated HCC. In this regard, a meta-analysis of 107 studies involving 19,319 cases of various malignancies and 25,675 controls demonstrated that a lower level of circulating adiponectin is associated with most types of cancer, but not HCC, in which the circulating adiponectin seems to be increased [70]. This was confirmed by a recent study using mendelian randomization analysis and genetic information in an East Asian population, which showed that an increase in circulating adiponectin levels may increase the risk of HCC [71]. Similarly, a recent meta-analysis of 13 studies including 1156 HCC patients (mostly patients with viral-associated HCC) and 2363 cancer-free controls (mainly healthy controls or patients with viral-related cirrhosis) reported a higher level of circulating adiponectin in HCC patients versus controls [72]. Notably, no difference in circulating adiponectin was detected between HCC patients and cirrhotic patients in a subgroup analysis. These results are in line with the above-mentioned observation of higher adiponectin in NASH-related cirrhosis, i.e., when the disease progresses [67]. Furthermore, in a retrospective study of 105 hepatitis C virus (HCV) patients (50 cirrhotic HCC, 19 non-cirrhotic HCC, and 36 cirrhotic without HCC) and 21 apparently healthy controls, circulating adiponectin was higher in cirrhosis and cirrhotic HCC patients compared with controls, but no difference was observed in the comparison between noncirrhotic HCC patients and controls [73], which further strengthened the previous hypothesis, although, again, this study was not referring to patients with NAFLD. However, given that approximately 20–25% of all NASH patients develop HCC in the absence of cirrhosis, it would be interesting to investigate more in-depth the role of adiponectin in non-cirrhotic NAFLD-associated HCC.

Furthermore, a higher circulating adiponectin seems to be related not only with an increased risk of HCC, but also with a poor survival of HCC patients based on the findings of a prospective study of 140 HCC patients, being followed-up for approximately 8 months [74]. Notably, in this study, 36% of participants had T2DM and probably NAFLD-associated HCC [74]. Contrary to circulating adiponectin, which seems to be increased in HCC, showing a positive association with HCC risk and a negative association with HCC survival in most clinical studies, data on the adiponectin expression in HCC tissue are conflicting. A study evaluating tissue specimens from 75 patients with surgically resected HCC with immunochemistry showed that adiponectin and AdipoR1 expression were lower in HCC tissue than in non-cancerous hepatic tissue [75]. Moreover, immunopositivity for adiponectin was correlated with smaller tumor size and better survival of HCC patients [75], which contrasts the findings of another study of similar design that showed the immunopositivity for adiponectin in HCC tissue was associated with a poor survival rate in HCC patients [76]. Considering all the above facts, we need, first, more case–control studies with specific populations of NAFLD-associated HCC to validate whether circulating adiponectin is higher in patients with than without NAFLD-associated HCC. Second, we need studies to evaluate the association between circulating and hepatic (in HCC and the adjacent non-cancerous tissue) adiponectin, so as to clarify whether circulating adiponectin appropriately reflects its hepatic concentrations. Third, we need prospective cohort studies to clarify whether baseline circulating adiponectin and/or hepatic (in HCC and the adjacent non-cancerous tissue) adiponectin may serve as positive or negative predictors of the survival in NAFLD-associated HCC.

## 4. Treatment Considerations for NAFLD-Associated HCC

Despite the increasing global prevalence of NAFLD and its associated prevalence of HCC, it seems paradoxical that there is not yet a medication particularly licensed for its treatment. Additionally, as obesity seems to increase the risk of HCC independent of NAFLD, targeting obesity through lifestyle modifications, pharmacological interventions, or even surgical approaches may be a rational strategy to prevent the progression of NAFLD, including the occurrence of HCC. Indeed, a meta-analysis demonstrated that bariatric surgery, known to attenuate metabolic diseases such as NAFLD and T2DM, clearly reduced the possibility of HCC (adjusted OR: 0.63; 95% CI: 0.53–0.75) [77].

When HCC occurs, modern treatment options vary and depend largely on tumor stage based on the BCLC system [6,78]. Unfortunately, only 25% of patients have early-stage disease at the time of diagnosis, when available treatments (hepatic resection, liver transplantation, and loco-regional therapies) may be curative [79]. On the contrary, the majority of patients present with late-stage disease, rendering systemic therapy to be the only treatment option for most of them. Tyrosine kinase inhibitors (TKIs) and immune checkpoint inhibitors (ICIs) are the mainstay of systemic therapy for advanced HCC. However, TKIs (sorafenib and lenvatinib as first-line options, and regorafenib, cabozantinib, and ramucirumab as second-line options) have demonstrated modest survival benefits, mainly due to resistance to treatment [80]. Over the last decade, ICIs have revolutionized the landscape of systemic therapies for liver cancer [81]. ICIs function by blocking two critical immune checkpoints: the cytotoxic T-lymphocyte-associated protein-4 (CTLA-4), which is expressed by T cells, and the PD-1 or PD-L1, which are expressed by T cells or tumor cells, respectively [82]. Since the overproduction of CTLA-4 and the activation of PD-L1/PD-1 system suppresses the immune response, their inhibition by ICIs have the potential to improve anti-tumor immunity, thus possibly providing more effective management of HCC [82].

**Figure 2:**
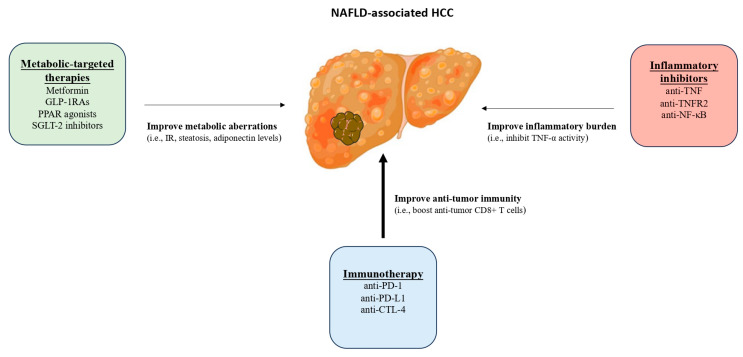
Combinational therapy for NAFLD-associated HCC. Emerging preclinical evidence suggests that ICIs, which are currently considered the standard of care for patients with advanced HCC, independent of the underlying etiology, may be less effective particularly in patients with NAFLD-associated HCC. It has been hypothesized that the dysmetabolic and inflammatory microenvironment in NAFLD and NAFLD-associated HCC, which potentially impacts the functionality of the immune system, may set the basis for repurposing metabolic-targeted therapies (therapies that target impaired metabolic pathways in NAFLD, such as IR, hepatic steatosis, adiponectin levels) or anti-inflammatory therapies (therapies against key inflammatory mediators, such as TNF-α) in combination with systemic immunotherapy against NAFLD-associated HCC. Abbreviations: CTL-4, cytotoxic T-lymphocyte-associated protein-4; HCC, hepatocellular carcinoma; ICIs, immune checkpoint inhibitors; IR, insulin resistance; GLP-1RAs, glucagon-like peptide-1 receptor agonists; NAFLD, nonalcoholic fatty liver disease; NF-κΒ, nuclear factor-kappa B; PPAR, peroxisome proliferator-activated receptors; PD-1, programmed death-1; PDL-1, programmed death-ligand 1; SGLT-2, sodium-glucose co-transporter-2; and TNF-α, tumor necrosis factor alpha.

However, preclinical evidence suggests that ICIs may be less effective against NAFLD-associated HCC compared with HCC of other etiologies. Specifically, in a mouse model of NASH-associated HCC, anti-PD-1 treatment not only failed to induce tumor regression, but also appeared to promote liver damage, a finding that was mechanistically linked to the increased activation of CXCR6+ PD1+ TNF+ CD8+ T cells [16]. As mentioned above, this subset of T cells seems to contribute to the progression of NAFL to NASH, and are distinct from T cells engaged in anti-tumor surveillance [15]. In this regard, the administration of ICIs in NAFLD-associated HCC intended to boost anti-tumor CD8+ T cells, but they may also inadvertently increase the number or activity of CXCR6+ PD1+ TNF+ CD8+ T cells, thus promoting the progression of NAFLD [17]. Consistent with this observation, some retrospective clinical data reinforce the notion that NAFLD-associated HCC may be less responsive to ICIs compared with viral-associated HCC [83], although conflicting results have been reported by other authors [84,85]. Thus, ICIs should be cautiously selected as the first-line therapeutic option for NAFLD-associated HCC, until future studies provide a clearer understanding of the efficacy of ICIs in NAFLD-associated HCC. Notably, it becomes more and more apparent that the dysmetabolic and inflammatory microenvironment in NAFLD and NAFLD-associated HCC may impact the functionality of the immune system [86,87,88]; therefore, the development of combination therapies that target both immune responses and metabolic or inflammatory factors (such as adiponectin or TNF-α or both) may hold a promise as a potential therapeutic approach for treating HCC in the context of NAFLD (Figure 2). In this regard, combination treatment of NAFLD, i.e., targeting more than one of its pathogenic contributors (e.g., metabolic, inflammatory, fibrogenic, and possibly tumorigenic) in an individual basis was long ago proposed by our group [89,90]. This may become more apparent in the light of the failure of most medications to meet their primary endpoints in clinical trials of NAFLD, implying that a single medication may not be suitable for all NAFLD patients due to the high heterogeneity of the pathogenesis of the disease [90]. Considering the above facts, this may be the case even in the NAFLD-associated HCC, in which metabolic and inflammatory factors may be targeted together with tumor-related factors.

In this regard, metabolic-targeted therapies (e.g., therapies against metabolic targets, such as IR, and hepatic steatosis) may assist the efficacy of systemic therapy against NAFLD-associated HCC. One example is metformin, an effective first-line medication for T2DM, but a non-recommended medication for NAFLD, as it largely failed to improve hepatic histology in biopsy-proven NASH [91]. Metformin exhibits multiple metabolic functions; it suppresses hepatic gluconeogenesis, thus decreasing plasma glucose levels [92], upregulates endogenous adiponectin, thus decreasing IR [93], and reduces de novo lipogenesis, thus possibly decreasing hepatic steatosis [94]. In addition, metformin reduced hepatic TNF-α in an experimental NASH model [95] and circulating TNF-α in T2DM patients with concomitant NAFLD, implying a potential anti-inflammatory action [96]. Metformin was recently investigated in mice with NASH-associated HCC in combination with anti-PD-1 treatment [97]. The authors confirmed previous findings regarding the reduced efficacy of ICIs against NAFLD-associated HCC; however, in this experimental study the addition of metformin to anti-PD-1 therapy was shown to reverse the low therapeutic efficacy of anti-PD-1, mainly by restoring the speed and motility of anti-tumor CD8+ T cells in NASH-associated HCC [97,98]. On the contrary, in a retrospective study of 397 patients with advanced HCC (of mixed etiologies) receiving immunotherapy (139 of them with T2DM and 80 on metformin), the use of metformin was not associated with improved survival [99]. However, the results of this study may have been affected by the higher proportion of patients with viral-associated HCC vs. NAFLD-associated HCC (72.7% vs. 9.8%). It should also be highlighted that the combination of metformin with TKIs may be challenging in the clinical setting, as a recent study of 279 patients with advanced HCC (86 of them with T2DM and 52 on metformin) reported that those treated with sorafenib and metformin had worse survival compared with those receiving only sorafenib; this unexpected finding was attributed to a higher resistance to sorafenib treatment caused by the chronic treatment with metformin [100]. Collectively, some, but not all, preliminary findings imply that metformin may be beneficial as adjuvant to ICIs in the treatment of NAFLD-associated HCC, even though it is a medication that failed to histologically improve NAFLD. This old and cheap antidiabetic medication may improve some metabolic aberrations in NAFLD, but also may modulate the immune microenvironment of HCC [101]; however, this remains to be proven in the future, using specifically designed clinical trials in patients with exclusive NAFLD-associated HCC. On the other hand, limited data do not support the combination of metformin with TKIs; although these data do not warrant the setting of clinical trials towards this aim, the use of metformin in patients with T2DM and concomitant HCC may be reconsidered in clinical practice, at least until more observational data are published on this topic.

Other medications in the pipeline for NASH may potentially increase the efficacy of systemic therapy, when used together for the treatment of NAFLD-associated HCC. Liraglutide, a glucagon-like peptide-1 receptor agonist (GLP-1RA) [102], was recently shown to facilitate anti-PD-1 treatment in Hepa1-6 tumor-bearing C57BL/6 mice, mainly by reducing the formation of neutrophil extracellular traps (NETs), although this was not a mouse model of NAFLD-associated HCC [103]. Notably, GLP-1RAs have demonstrated anti-steatotic and anti-inflammatory properties (partly attributed to their adiponectin-increasing [104] and TNF-α-reducing effect [96]), albeit not anti-fibrotic properties [105,106,107]; thus, they may likely act in conjunction with ICIs in NAFLD-associated HCC. Obviously, additional mechanistic studies are required to clarify any potential additive or synergistic effects of GLP-1RAs and ICIs, particularly in regard to NAFLD-associated HCC. Notably, monotherapy with liraglutide [108] or exenatide [109], another GLP-1RA, was shown to prevent the development of HCC in NASH mouse models. Similarly, lower rates of HCC were observed in T2DM patients on GLP-1RAs compared with those on insulin, although this was a secondary endpoint in a retrospective cohort study [110]. These findings warrant further research on the use of GLP-1RAs as a preventive strategy for the onset of NAFLD-associated HCC, as well as enhancers of the efficacy of ICIs in NAFLD-associated HCC. Experimental evidence also suggests that saroglitazar, a dual PPAR-α/γ agonist [111], and canagliflozin, a sodium-glucose co-transporter-2 (SGLT-2) inhibitor [112], may prevent the onset of NAFLD-associated HCC. Importantly, saroglitazar was shown to increase adiponectin and decrease TNF-α in a rat model of NASH [113]. Remogliflozin, a SGLT-2 inhibitor, was shown to reduce hepatic TNF-α expression in a HFD model of NAFLD [114], and dapagliflozin, another SGLT-2 inhibitor, was shown to increase circulating adiponectin in a study with T2DM patients with NASH [115]. Based on these initial findings, saroglitazar and SGLT-2 inhibitors, which are under investigation for the management of NAFLD [116,117], require further research as add-on ICIs or TKIs in patients NAFLD-associated HCC.

In contrast to the above-mentioned medications that target multiple parallel metabolic pathways (including the production and secretion of endogenous adiponectin and/οr inhibition of TNF-α, at least in part), the direct anti-tumoral potential of adiponectin is more difficult to be investigated. The production of functionally active recombinant adiponectin has been proven to be extremely challenging, since adiponectin is secreted in complex multimers and undergoes extensive post-translational alterations before its secretion by the adipocytes, thereby rendering the development of fully functional recombinant adiponectin to be demanding [118,119]. Alternatively, small molecules functioning as adiponectin receptor agonists (e.g., osmotin and AdipoRon) may be tested for NASH and NAFLD-associated HCC in the near future [54,120].

Besides metabolic-targeted therapies, combination treatment, including inhibitors of mediators of inflammation (e.g., anti-TNF), and systemic therapy may also be a rational strategy against NAFLD-associated HCC. Currently, the blockade of TNF signaling with infliximab is recommended for the treatment of serious diseases (e.g., colitis and hepatitis) associated with immunotherapy [121]. Interestingly, the administration of anti-TNF (infliximab or etanercept) in combination with anti-CTLA-4 and anti-PD-1 immunotherapy reduced serious adverse events (i.e., colitis) and, also, improved anti-tumor efficacy in experimental mouse models of colon cancer and melanoma [122]. Although anti-TNF therapy in cancer treatment is still a controversial topic, due to the complex role of TNF-α and NF-κΒ in cancer pathogenesis [123], the above experimental studies suggest that anti-TNF biologics combined with ICIs may potentially enhance the efficacy and ameliorate the side effects of the latter. Nevertheless, studies evaluating the combined effect of anti-TNF and ICIs in NAFLD-associated HCC remain scarce. There are only hints obtained from a recent mouse model of NASH-associated HCC that suggest that TNF-α neutralization may prevent anti-PD-1 exacerbation of HCC, which, however, warrants further mechanistic studies [16]. Previous preclinical studies testing anti-TNF agents in xenograft HCC [124] or chemically induced HCC mouse models [125], but not in obesity- or NASH-associated HCC mouse models, demonstrated that the blockade of TNF-α signaling hampered tumor progression by diminishing the expression of proinflammatory mediators within HCC tissue and promoting apoptosis in HCC cell. In addition, the combination of anti-TNF with 5-fluorouracil (FU), a classic chemotherapeutic agent, also inhibited tumor growth and improved survival of a mouse model of xenograft HCC [44]. It is also worth noting that, unlike metformin, anti-TNF seem to potentiate the efficacy of sorafenib [126], which, if validated in clinical trials, may also be important in the clinical setting, as sorafenib is also considered a first-line option for HCC. However, the combined effect of anti-TNF and ICIs should be more extensively evaluated in preclinical models of NASH-associated HCC to guide its potential use in relevant clinical trials. Given that higher TNF-α levels are probably associated with a worse survival in patients with advanced HCC, but not early HCC [41,44,45,47], the use of anti-TNF may prove beneficial, especially for the subgroup of patients with advanced NASH-associated HCC, which, however, remains to be elucidated in future studies.

Interestingly, as mentioned above, TNFR2 has also attracted considerable attention for its emerging role in carcinogenesis and tumor progression, and it is under evaluation as a novel strategy in cancer immunotherapy [127]. New generation medications that selectively target TNFR2, but not TNFR1, may prove to be more suitable as adjuvants to ICIs than anti-TNF agents, which, however, may be elucidated in the future. Therefore, the upstream inhibition of the TNF pathway (e.g., anti-TNF agents or anti-TNFR2) or the selective suppression of NF-κΒ in immune cells rather than ubiquitous NF-κB inhibition, if applicable, may possibly be a more targeted and effective management against NASH-driven HCC.

## 5. Conclusions and Future Directions

TNF-α, a key proinflammatory cytokine, and adiponectin, a key adipokine, are implicated in the pathogenesis of NAFLD and possibly in the progression to advanced disease, including NAFLD-associated HCC, which is supported by most of the current experimental and limited observational studies [8,9,10]. Noteworthily, their presumable contribution to NAFLD-HCC bears important clinical implications and therapeutic considerations that warrant further evaluation in future studies.

A critical research objective is to identify easily measurable serum or plasma biomarkers that may assist clinicians with the early diagnosis, staging, and prognosis of patients with NAFLD-associated HCC, which remains an unmet clinical need. Different panel of circulating molecules (e.g., cytokines, adipokines, chemokines, and growth factors) are associated with different mechanisms of carcinogenesis (e.g., angiogenesis, chronic inflammation, fibrosis, and tumor invasiveness) [128]. In this regard, some cytokines may predict an HCC stage more accurately than others, i.e., cytokines that have predictive value for one HCC stage may not be relevant for other HCC stages [129]. TNF-α stimulates chronic inflammation and seems to be progressively increased during the progression from NAFLD to HCC; however, whether TNF-α plays a more significant role in early stages (i.e., development of HCC) or advanced stages (i.e., progression of HCC) remains uncertain. There is also evidence suggesting that a higher hepatic expression of TNF-α in HCC tissue may be associated with a better prognosis in early stage HCC, but with a worse prognosis in late-stage HCC [44,45,47]. However, this may not be relevant for circulating TNF-α, which did not help to differentiate between cirrhotic patients with from those without HCC in one study [41]. More studies evaluating the role and utility of circulating, hepatic, and tumor TNF-α, specifically in patients with NAFLD-associated HCC, are warranted in the future to better define the association between circulating TNF-α with hepatic TNF-α from both malignant and healthy hepatic tissues. For adiponectin, it is initially decreased, but paradoxically increased at end stage disease, i.e., cirrhosis and HCC. Limited clinical evidence suggests that a higher circulating adiponectin is associated with an increased risk and a poor survival of HCC patients [74]. Obviously, more case–control studies with specific populations of NAFLD-associated HCC are needed to validate whether circulating adiponectin is higher in patients with than without NAFLD-associated HCC. In addition, prospective cohort studies to clarify whether baseline circulating adiponectin may serve as a predictor of the survival in NAFLD-associated HCC are also warranted.

In addition, given their putative role in the pathogenesis of NAFLD and its progression to HCC, TNF-α and adiponectin, which seem to antagonize each other, are potentially promising therapeutic targets to be evaluated in the setting of NAFLD-associated HCC, alone or, even better, in combination with systematic therapy, e.g., ICIs, which seem to be less effective in NAFLD-associated HCC than in non-NAFLD-HCC [16,83]. This appears more feasible today for TNF-α, since anti-TNF biologics have been approved for inflammatory and rheumatologic diseases. Currently, anti-TNF agents (e.g., infliximab) are recommended for the treatment of immunotherapy-related serious adverse events (e.g., colitis and hepatitis). Notably, emerging experimental evidence suggests that anti-TNF may also boost the efficacy of ICIs, as well as TKIs; therefore, their combined effect should be more extensively evaluated in preclinical models of NASH-associated HCC to guide their potential use in relevant clinical trials.

Regarding adiponectin, the use of recombinant adiponectin is limited due to its complex isomers and its extensive post-translational modifications that are required to be fully functional; however, there are medications such as small adiponectin analogs that activate adiponectin receptors, which may prove clinically useful [54,120]. Additionally, medications in the pipeline for NASH, including GLP-1RAs and PPAR modulators can augment the production of endogenous adiponectin and decrease TNF-α concentrations, being potentially beneficial as add-on to ICIs. Obviously, there is a clear need for further mechanistic studies to clarify any potential synergistic or additive effect of these medications with ICIs, particularly with regard to NAFLD-associated HCC. In case of encouraging results in mechanistic studies, observational studies, involving patients with obesity and/or T2DM and concomitant HCC on GLP-1RAs and/or PPAR modulators, may be the rational next step to evaluate the association between the specific anti-diabetic medications with HCC. Last but not least, as both obesity and NAFLD may lead to the development of HCC, and in the absence of any approved medication specifically for NAFLD, it should be evaluated whether targeting obesity may prevent NAFLD-associated HCC, by affecting adiponectin and/or TNF-α production.

Considering all the above facts, TNF-α and adiponectin seem to participate in the progression of NAFLD to advanced disease and HCC, a finding that renders both of them as potential diagnostic biomarkers and appealing therapeutic targets for the management of NAFLD-associated HCC.

## Data Availability

No raw data were generated for the needs of this narrative review.
